# Abstract of Meteorological Observations for February, 1854, Made at Philadelphia, Pa.

**Published:** 1854-04

**Authors:** James A. Kirkpatrick


*Abstract of Meteorological Observations for February,* 1854, *made at Philadelphia, Pa. By* PROF. JAMES A. KIRKPATRICK.

| 1854. Feb. | BAROMETER. Mean. | THERMOMETER. Mean. | THERMOMETER. Daily Range. | Dew point 2 P. M. | Prevailing Winds. | General Remarks. |
|---|---|---|---|---|---|---|
| | Inches. | deg. | deg. | deg. | Points. | |
| 1 | 29.553 | 45.0 | 18 | 35.3 | S.W. | M. and aft. cloudy, ev. clear. |
| 2 | 29.510 | 51.0 | 20 | 44.7 | S.W. | M. & aft. cloudy, ev. clear, Therm. highest 61°, Barometer lowest 29 432. |
| 3 | 29.899 | 21.0 | 8 | 25.0 | N.W. | M. *Snow*, e. cloudy, a. clear. |
| 4 | 30.172 | 21.0 | 8 | 25.0 | N.W. | Clear. |
| 5 | 29.955 | 27.5 | 23 | 29.7 | (Var.) | M&a c'y, e. clear, T. low. 16° |
| 6 | 30.237 | 23.0 | 12 | 29.0 | N.W. | Clear. |
| 7 | 30.316 | 27.0 | 12 | 30.7 | (Var.) | Cloudy. |
| 8 | 29.691 | 39.5 | 20 | 46.7 | (Var.) | do. *Rain* all day. |
| 9 | 29.684 | 40.0 | 10 | 27.7 | S.W. | Clear. |
| 10 | 29.879 | 39.0 | 14 | 25.0 | (Var.) | do. |
| 11 | 30.243 | 25.7 | 11½ | 30.3 | N. W. | M. and aft. clear, ev. cloudy. |
| 12 | 30.254 | 30.0 | 10 | 35.0 | (Var.) | Cloudy. |
| 13 | 30.008 | 39.5 | 15 | 44.7 | N.E. | do. aft. and. ev. *Rain.* |
| 14 | 29.766 | 51.0 | 18 | 55.3 | N.E. | do. ev. *Rain.* |
| 15 | 29.744 | 42.0 | 6 | 42.7 | N. | do. light *Rain* all day. |
| 16 | 29.921 | 35.0 | 6 | 35.7 | S.W. | Morn. *Snow*, ev. clear. |
| 17 | 30.214 | 30.0 | 8 | 29.3 | W. NW. | M. and ev. clear, aft. cloudy. |
| 18 | 30.092 | 33.0 | 16 | 24.7 | W. S.W. | Clear. |
| 19 | 29.939 | 38.0 | 10 | 31.3 | N.W. | M. and ev. clear, aft. cloudy. |
| 20 | 29.817 | 28.0 | 6 | 31.0 | E. | Cloudy, *S* stm. 10½AM. began. |
| 21 | 29.751 | 30.0 | 14 | 27.7 | N.W. | M.c'y.*S* stop. 9½AM. e. clear. |
| 22 | 29.814 | 34.0 | 16 | 35.0 | S. W. | M. clear, aft. and ev. cloudy. |
| 23 | 30.000 | 22.0 | 6 | 25.0 | N.W. | Clear. |
| 24 | 30.112 | 26.5 | 17 | 28.0 | S.W. | do. |
| 25 | 30.310 | 32.0 | 4 | 27.0 | N.E. | M. clear, aft. andev. cloudy. |
| 26 | 29.644 | 41.0 | 20 | 48.7 | (Var.) | Cl'y, *Rain* till 4 PM. then fog. |
| 27 | 29.943 | 37.0 | 10 | 27.3 | N.W. | M. *R&S* till 9, a. c'y. e. c'r. |
| 28 | 30.319 | 31.0 | 12 | 27.7 | (Var.) | M& e. c'r, a. c'y, B. h. 30.404 |
| Monthly Mean 1854 | 29.957 | 33.4 | 45 | 33.0 | NW.SW | 4.20 in. rain and melt. snow. |
| Monthly Mean 1853 | 29.946 | 36.0 | 44½ | 35.8 | NW. W | 4.44 do do |
| Monthly Mean 1852 | 29.861 | 33.0 | 48½ | 30.4 | W. NW | 2.71 do do |
| Monthly mean for 3 years. | 29.921 | 34.1 | 46 | 33.1 | NWWSW | 3.78 inches. |

